# The rise of point-of-care genetics: how the SARS-CoV-2 pandemic will accelerate adoption of genetic testing in the acute setting

**DOI:** 10.1038/s41431-021-00816-x

**Published:** 2021-02-15

**Authors:** John H. McDermott, John Burn, Dian Donnai, William G. Newman

**Affiliations:** 1 Manchester Centre for Genomic Medicine, St Mary’s Hospital, Manchester University NHS Foundation Trust, Manchester, M13 9WL UK; 2 Division of Evolution and Genomic Sciences, School of Biological Sciences, University of Manchester, Manchester, UK; 3 Institute of Translational and Clinical Research, Newcastle University, Newcastle upon Tyne, NE1 7RU UK; 4 QuantuMDx Group Ltd, Lugano Building, 57 Melbourne St, Newcastle upon Tyne, NE1 2JQ UK; 5 The Newcastle upon Tyne Hospitals NHS Foundation Trust, Newcastle upon Tyne, NE7 7DN UK

**Keywords:** Genetic testing, Diagnostic markers

The SARS-CoV-2 outbreak has necessitated innovation in many areas, including the development of molecular point-of-care tests (POCTs) to facilitate rapid diagnosis. Academia and industry have collaborated to produce these diagnostics at an unparalleled pace, drawing on various methodological approaches. This effort is immediately important in controlling the pandemic, but we argue that it will also create a landscape where near-patient diagnostics become increasingly viable as an approach to rapidly test for human genetic variation. This could result in a new testing paradigm, where genotype is used to routinely tailor management. We make the case that the clinical genetics community is well-placed, given its unique history, to play a central role in the development and implementation of any new testing model.

Since the emergence of clinical genetics as a distinct specialty in the mid-20th century, significant technological innovations have often preceded major clinical breakthroughs [1]. In the late 1950s, cytogenetics emerged as an increasingly relevant diagnostic tool, facilitating the characterisation of many chromosomal disorders. Early practitioners of clinical genetics were no longer restricted to making diagnoses based on phenotype alone, rather they set about developing a specialty, which was able to call upon an ever-growing arsenal of molecular assays to complement their clinical acumen.

In the following decades technological advances such as Sanger sequencing led to the identification of hundreds of Mendelian disorders. Geneticists learned to integrate testing into their clinical process and became skilled at interpreting results in the context of a patient’s phenotype. The advent of Next Generation Sequencing expanded the ‘Mendeliome’ yet further, catalyzing the molecular classification of thousands of rare disorders. The ability to sequence an individuals’s genome has revolutionized the specialty, increasing the likelihood of a diagnosis, and expanding opportunities for research in those patients without one [2].

The concept of point-of-care genetic testing is not new but there are few instances of successful implementation into routine clinical practice. One of the first examples was the Cepheid’s GeneXpert assay designed to identify the BCR-ABL gene fusion [3]. Other companies have since developed rapid assays for use in clinical practice to determine an individual’s genetic-metaboliser status for several commonly prescribed medications [4, 5]. However, sustained clinical interest in these technologies has been limited, meaning industry resources are often concentrated elsewhere within their portfolio. Because of the COVID-19 pandemic, many companies have heavily re-focused efforts back towards their POCT systems, repurposing them to detect SARS-CoV-2.

SARS-CoV-2 is a single-stranded RNA virus, and gold-standard testing is via rRT-PCR. This typically requires resources and expertise embedded within a laboratory, meaning that the time between sample collection and a result can be well over 24 h, a delay which can result in inaccurate cohorting of patients with and without the virus. High-quality POCTs for SARS-CoV-2 could improve triaging accuracy and, in the UK, a national consortium has been formed to validate these tools given the complexity and regulatory challenges of implementing a novel in vitro device [6, 7].

Developing a clinically useful SARS-CoV-2 POCT could also have significant financial reward, therefore many companies are attempting to address this challenge, each bringing their own scientific perspective, technological solutions and intellectual property. This has resulted in a cornucopia of POCTs coming to market, which utilize variable approaches beyond standard PCR, including isothermal nucleic acid amplification, CRISPR-based diagnostics and NGS systems, such as the recently announced LamPORE [8, 9]. The success of these technologies will be determined by the ability to generate reliable results whilst allowing substantial multiplex capability, ensuring maximum clinical utility.

There is understandable uncertainty around the future trajectory of the pandemic. Assuming the effective roll out of efficacious vaccines, the prevalence of COVID-19 should decrease significantly [10]. As this begins to happen, SARS-CoV-2 testing will remain important, but it is highly likely that the quantity of tests performed will fall considerably. As market demand drops, one expects that companies will want to re-purpose these testing innovations, forged through monumental effort and considerable expense, towards other applications. Molecular diagnosis of other pathogens, such as sexually transmitted diseases or tuberculosis, will be a primary target. However, with minor adaptation, many of these systems will also be able to detect human variation.

There are a small but growing number of examples where rapidly identifying genetic variation is clinically relevant. The majority of these are related to pharmacogenetics, where awareness of a variant can be used to guide prescribing, improving the efficacy of treatment or avoiding adverse drug reactions. Rapid identification of genetic variation using POCTs could be used to guide antiplatelet therapy (*CYP2C19*), help tailor opioid-prescribing (*CYP2D6*), or could be used to avoid aminoglycoside-induced hearing loss (m.1555A>G) [5, 11].

The expansion of POCTs during the pandemic will result in a more mature and varied technological landscape, but it will also serve to normalise the use of these systems within everyday clinical practice. Most medical specialties do not routinely test for or interpret genetic variation, especially in the acute setting. Growing use of SARS-CoV-2 POCTs, coupled with an increased interest from industry, provides a fertile environment for the development and implementation of other near-patient genetic tests in acute healthcare settings.

Traditionally, clinical genetics services have been gatekeepers to genetic testing. The current POCT revolution may well change that. We believe that our community has a critical role to play in shaping this revolution and we must not be bystanders. By collaborating with industry to develop robust diagnostics and working alongside clinicians to integrate these tools into clinical pathways, we can help straddle the clinical–academic divide. The history of our specialty is, to a great extent, the history of molecular diagnostics. The rise of the genetic POCT is the next chapter in that story and we must adapt once more.
